# Supplementary figures and images for: Cave adaptation drives coordinated transcriptional remodeling across diverse cell types in the brain of a teleost fish
Source: bioRxiv. 2026 Jun 24:2026.06.19.733352. Preprint. [Version 1] doi: 10.64898/2026.06.19.733352 (PMC13320961; doi:10.64898/2026.06.19.733352)

leiden

UMAP2

UMAP1

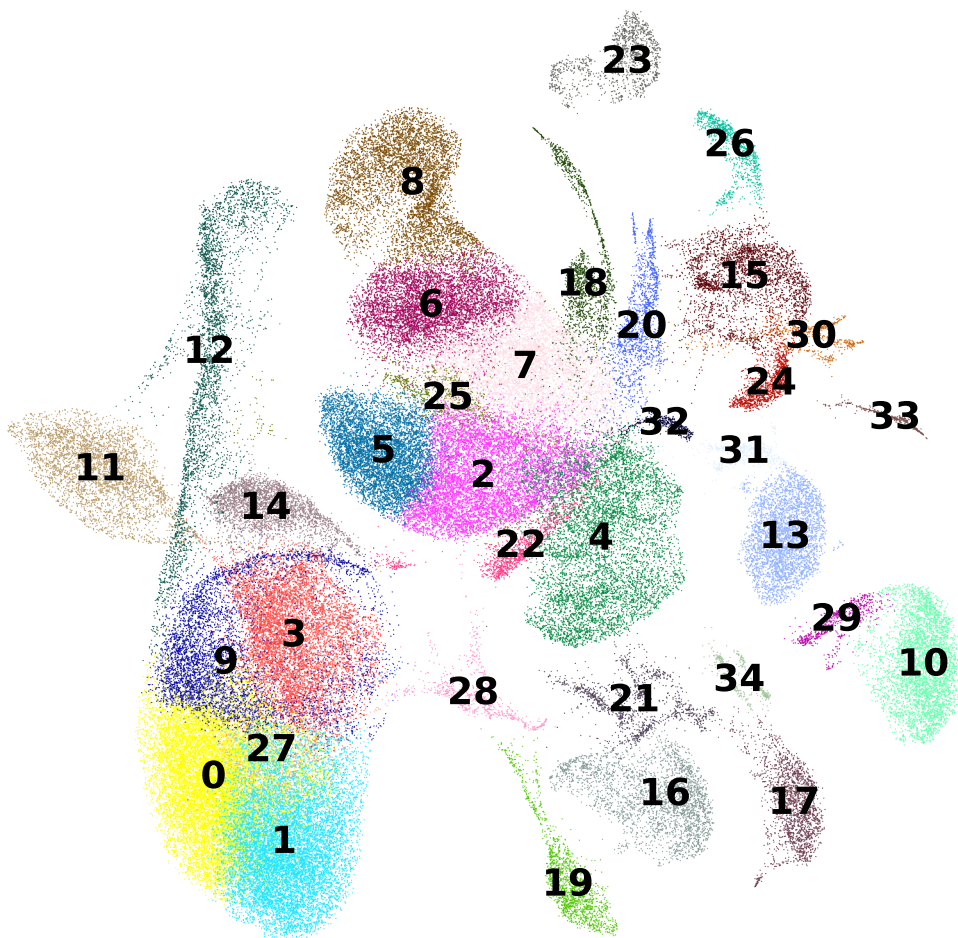

Supplement: Supplement 1 — Supplementary Figure 1. UMAP of full snRNAseq dataset, with clusters labeled by number according to size, with 0 the largest cluster and 35 the smallest cluster. [file media-1.pdf]

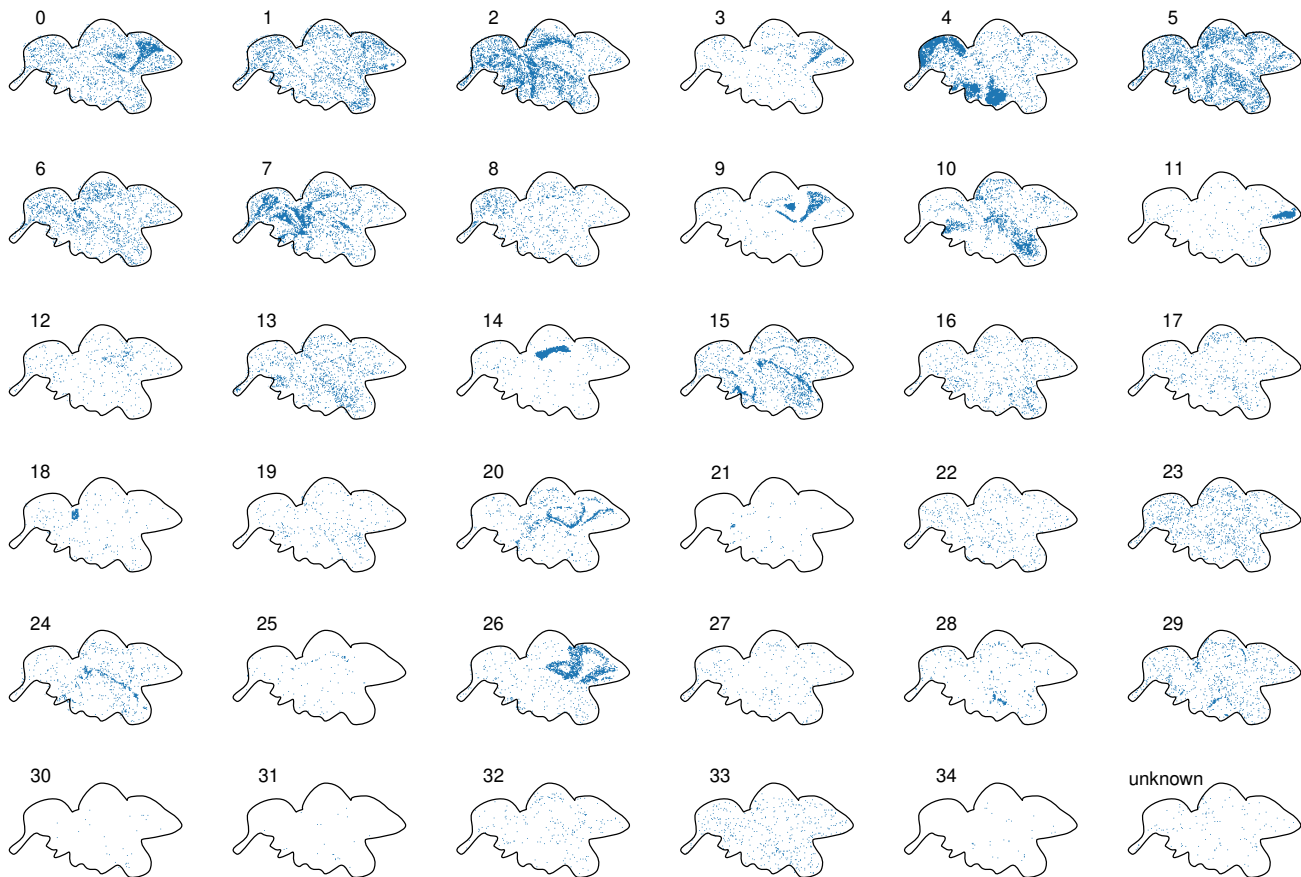

Supplement: Supplement 2 — Supplementary Figure 2. Spatial barcodes assigned to each snRNA cluster. [file media-2.pdf]

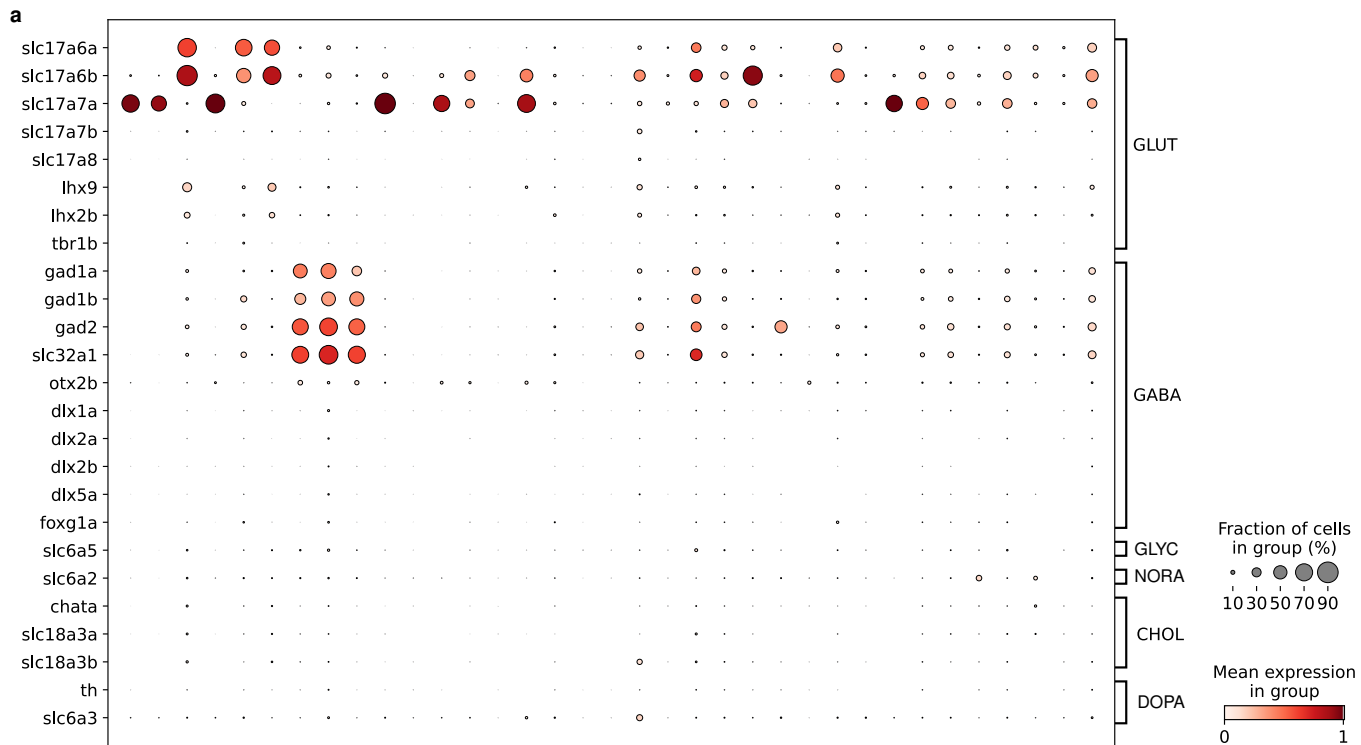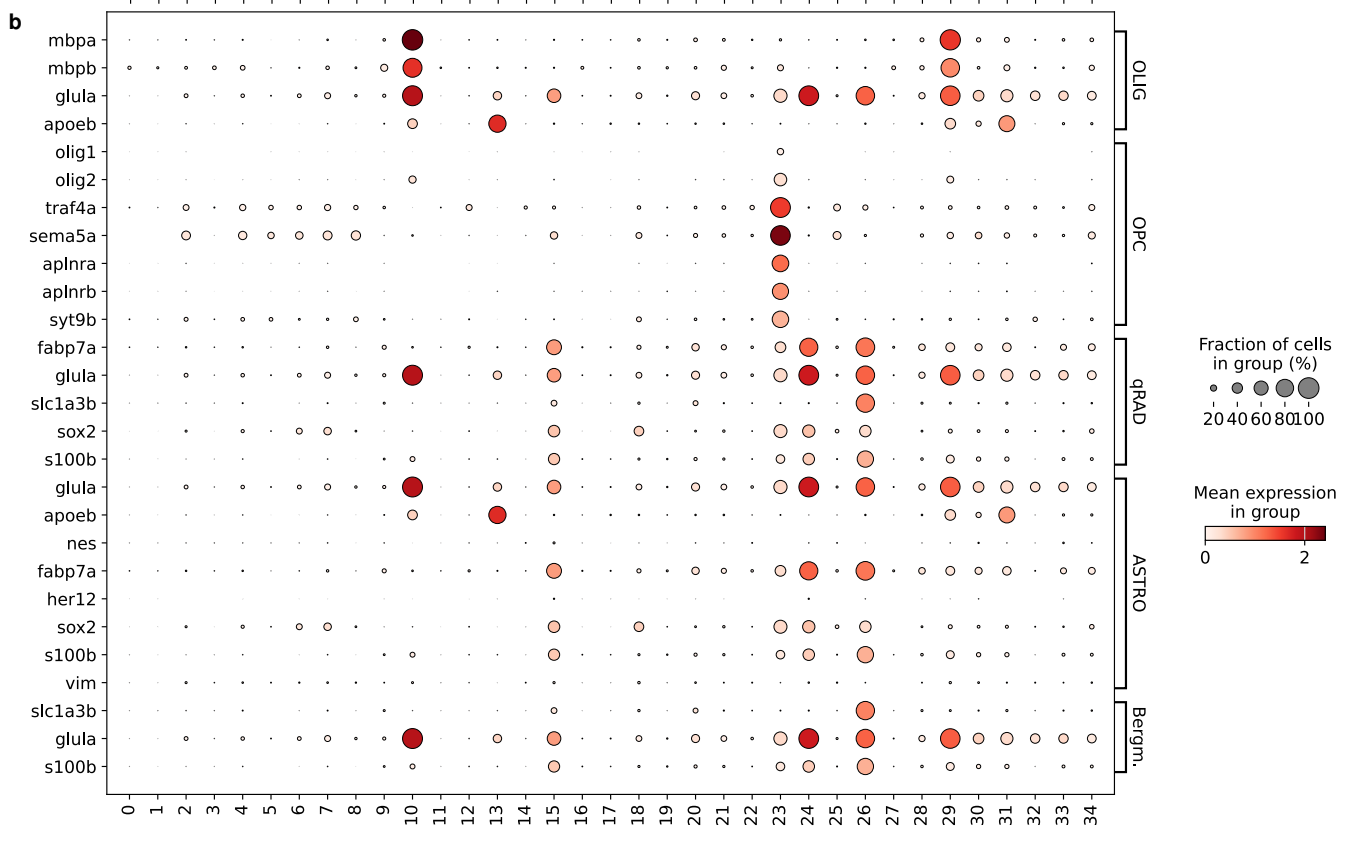

Supplement: Supplement 3 — Supplementary Figure 3. Marker gene expression for neurons (a) and glia (b). [file media-3.pdf]

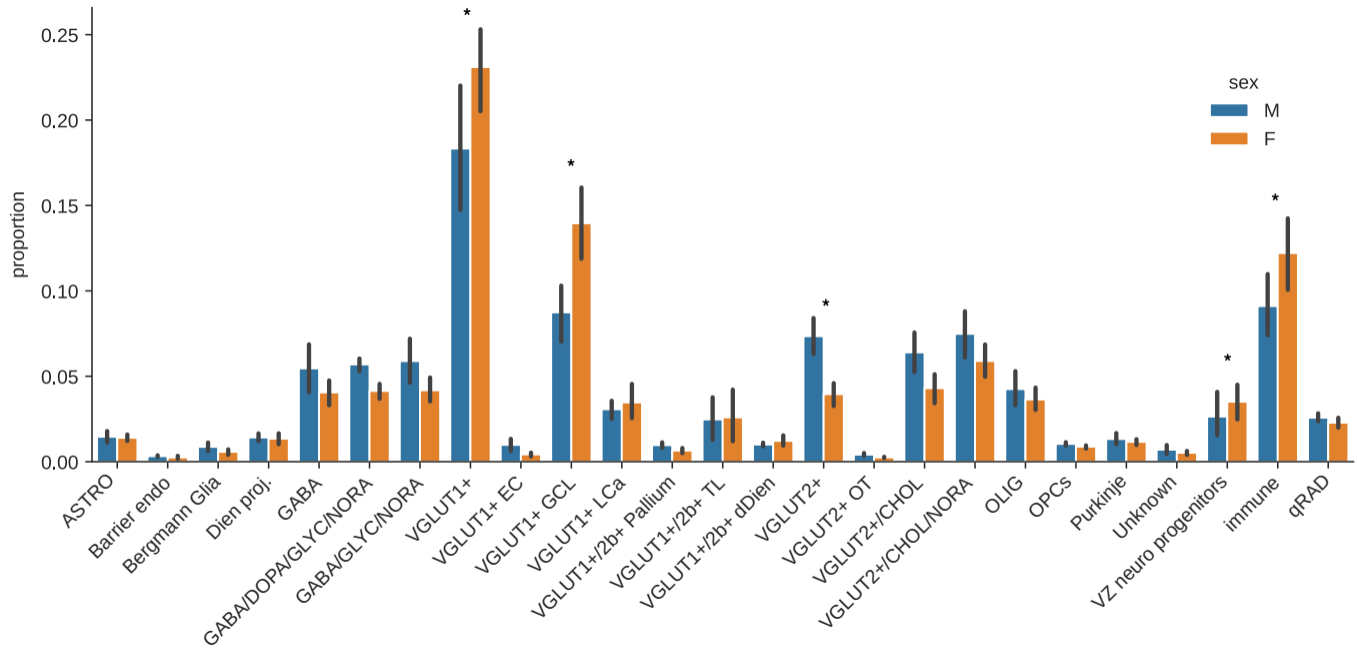

Supplement: Supplement 4 — Supplementary Figure 4. Cell type composition by sex. Clusters for which composition is different between male and female samples at FDR = 0.1 are denoted by *. 95% confidence intervals are shown. [file media-4.pdf]

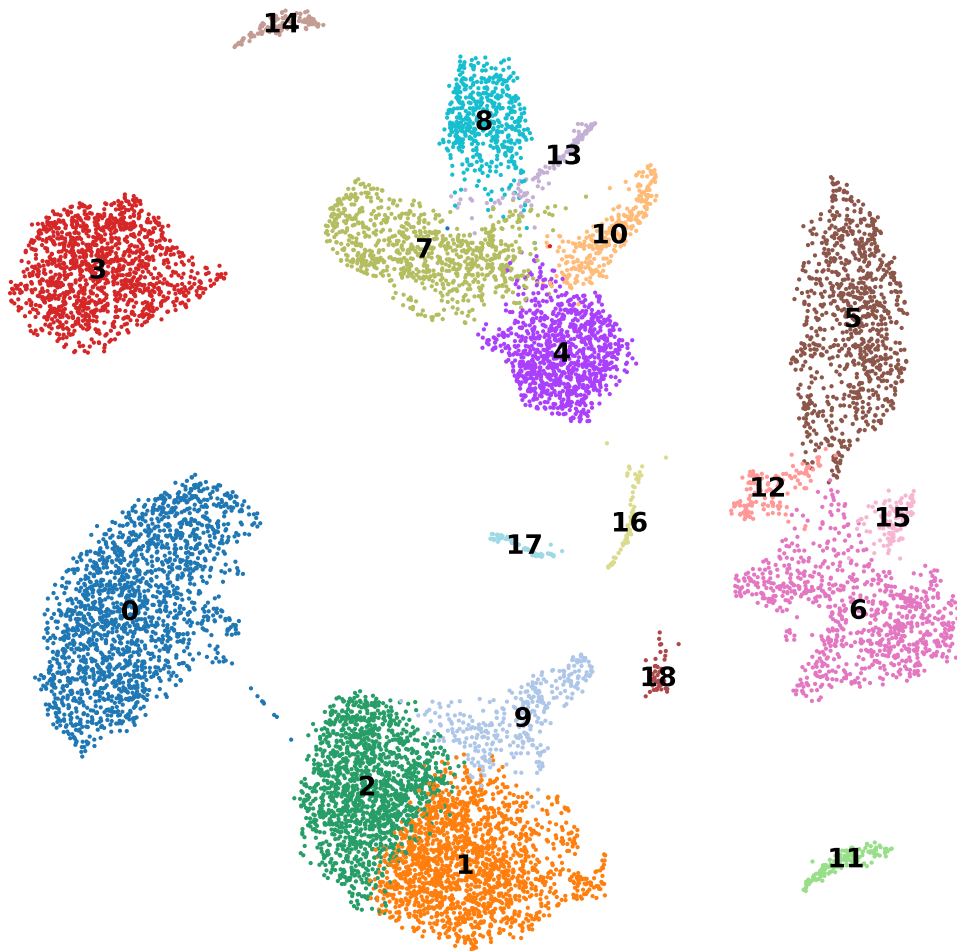

Supplement: Supplement 5 — Supplementary Figure 5. UMAP of immune subclusters, with clusters labeled by number according to size. [file media-5.pdf]

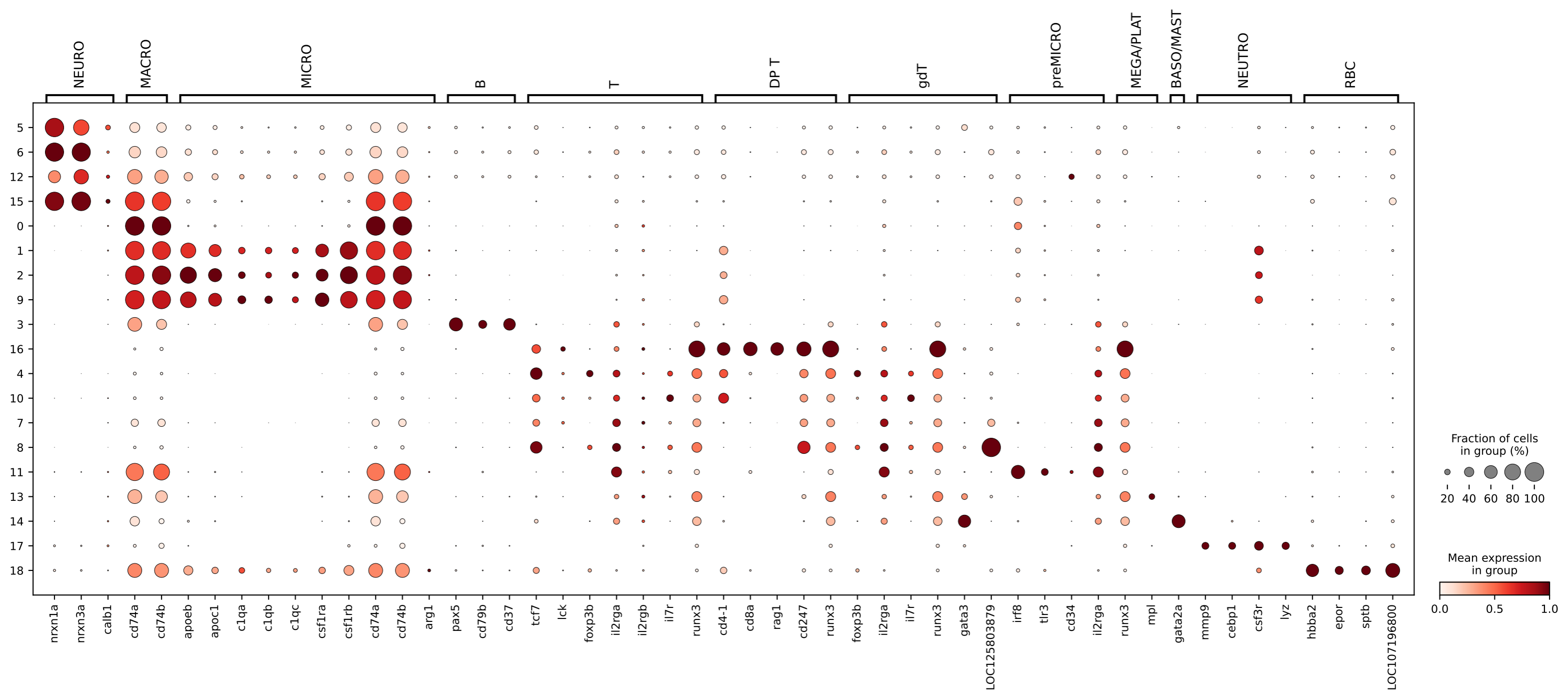

Supplement: Supplement 6 — Supplementary Figure 6. Marker gene expression for subclustered immune cells. [file media-6.pdf]

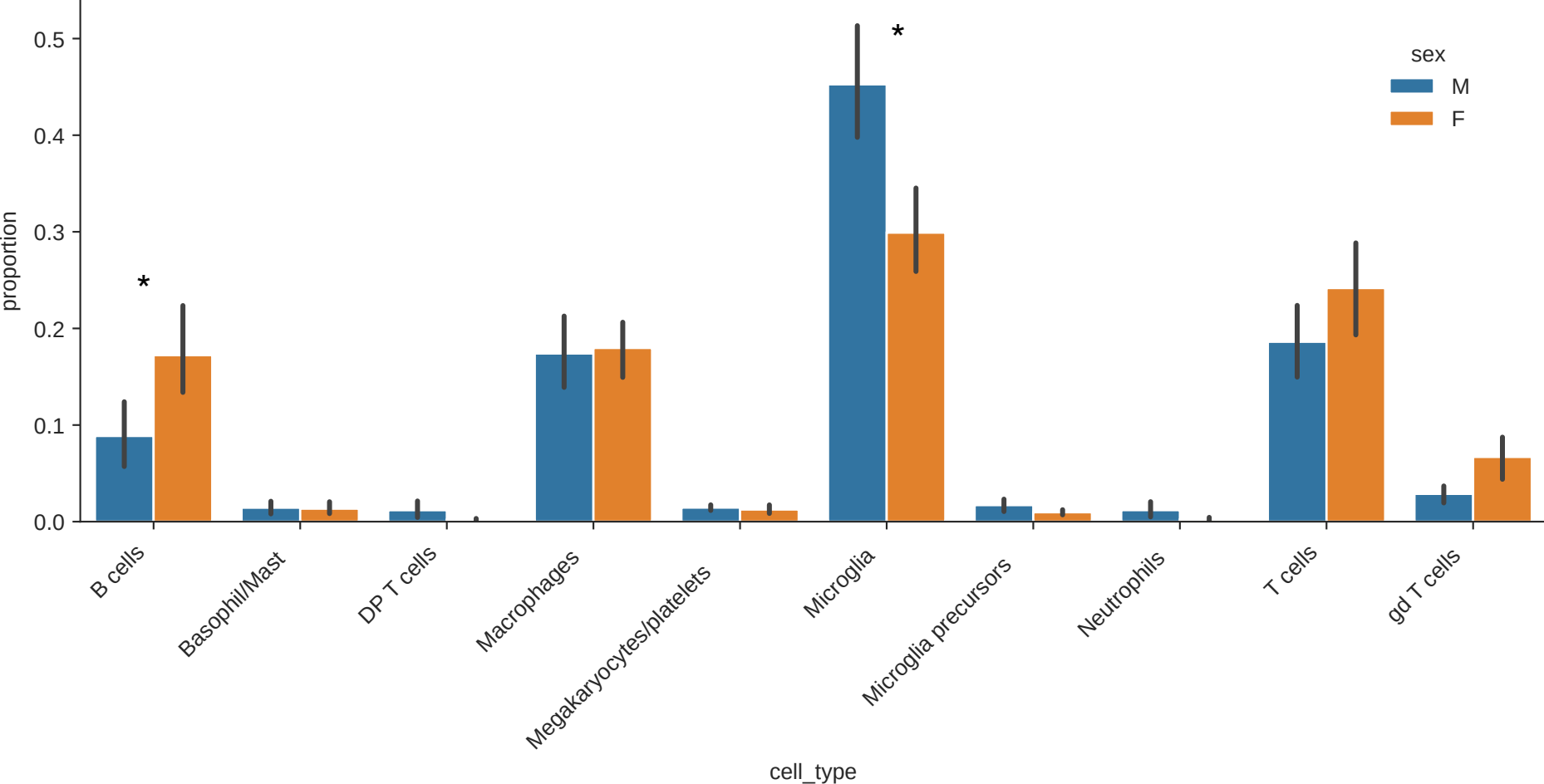

Supplement: Supplement 7 — Supplementary Figure 7. Cell type composition for immune cells by sex. Clusters for which compositions is different between male and female samples at FDR = 0.05 are denoted by *. 95% confidence intervals are shown. [file media-7.pdf]
